# Supplementary material for: Thermoelectric Signal Enhancement by Reconciling the Spin Seebeck and Anomalous Nernst Effects in Ferromagnet/Non-magnet Multilayers
Source: Sci Rep. 2015 May 28;5:10249. doi: 10.1038/srep10249 (PMC4447118; doi:10.1038/srep10249)
Supplement: Supplementary Information [file srep10249-s1.pdf]

Supplementary Information

Thermoelectric Signal Enhancement by Reconciling the Spin Seebeck and Anomalous Nernst Effects in Ferromagnet/Non-magnet Multilayers

Kyeong-Dong Lee[1], Dong-Jun Kim[1], Hae Yeon Lee[1], Seung-Hyun Kim[2], Jong-Hyun Lee[2], Kyung-Min Lee[2], Jong-Ryul Jeong[2], Ki-Suk Lee[3], Hyon-Seok Song[4,5], Jeong-Woo Sohn[4,5], Sung-Chul Shin[4,5], and Byong-Guk Park[1*]

[1]*Department of Materials Science and Engineering, KI for the Nanocentury, KAIST, Daejeon, 305-701, Korea*

[2]*Department of Materials Science and Engineering, Graduate School of Green Energy Technology, Chungnam National University, Daejeon, 305-764, Korea*

[3]*School of Mechanical and Advanced Materials Engineering, UNIST, Ulsan, 689-798, Korea*

[4]*Department of Physics and CNSM, KAIST, Daejeon, 305-701, Korea*

[5]*Department of Emerging Materials Science, DGIST, Daegu, 711-873, Korea*

*Email: bgpark@kaist.ac.kr

Spin thermoelectric voltage (ΔV) dependence on laser power and its spot size. We observe that the thermal voltage does not depend on the beam size (d) between 10 and 330 μm for the sample w = 400 μm under the same laser power (P), as shown in Fig. S1. The temperature gradient is well-defined with P, which is evidenced by the fact that the ΔV shows linear tendency with P, except a deviation for a very high power density. Note that our beam size is defined experimentally from the 10-90% reflectivity change when the laser beam moves across the edge boundary of a photo-lithographically patterned stripe structure. Rewriting the equation (2) in the main text leads to $V_{ANE} \approx (C_{ANE}M_s w^{-1})(d^2\nabla T_F)$, when $R_F << R_N$. When $R_F >> R_N$, $V_{SSE} \approx -(S_S w^{-1})(d^2\nabla T_{FN})$. Since d is changing while V remains constant, the $d^2\nabla T$ is thought to be conserved under the same laser power and sample condition. This implies that the power density ($\sim Pd^{-2}$) is proportional to the temperature gradient of ∇T_F and ∇T_{FN}. In other words, in the linear regime of optical energy transfer to heat,

$$V \times wP^{-1} \propto S_S \text{ or } C_{ANE}M_s, \qquad \text{(S1)}$$

This could be very useful when we calibrate the calculated temperature to fit the experimental condition, or when we compare the measured signal with the literature value, measured usually with different beam size and power. Under the same P condition, we used Gaussian beam radial size (σ_L) of 31.2 μm in the calculation, whereas the actual beam diameter (10-90% criteria ~ 2.564σ_L) was 80 μm. Here, we matched σ_L to reflect the actual power density correctly with the calculated one $((80\ \mu m/2.564) / 31.2\ \mu m)^2 \sim 1$. This is considered in the calculation of C_{ANE} and S_S in equation (2) of the main text.

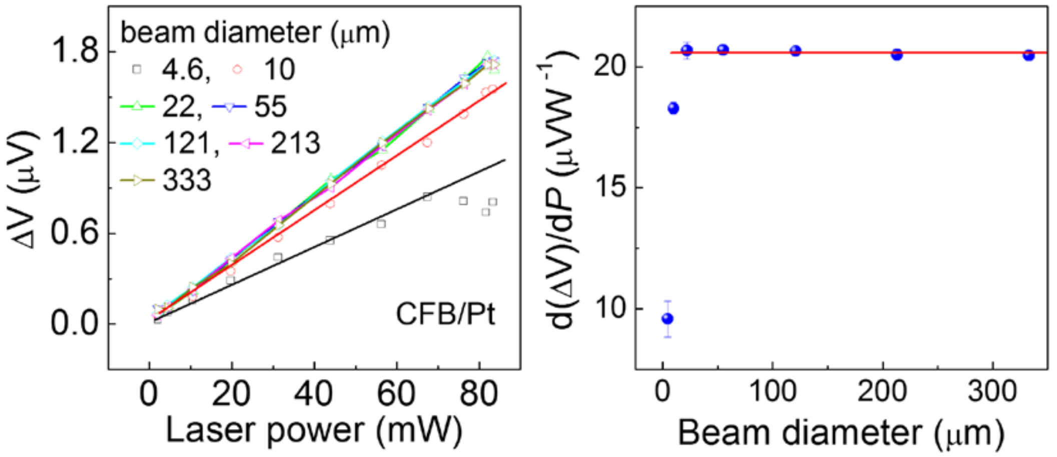

Figure S1 | Spin thermoelectric voltage dependence of CoFeB/Pt (w = 400 μm) on laser power (left) and its spot size (right panel).

The effect of normal Seebeck effect. Normal Seebeck effect might be involved in the process of charge accumulation. However, from five reasons, we cannot explain the measured data only by normal Seebeck effect without considering the inverse spin Hall effect. 1) In our experimental configuration of longitudinal geometry, vertical thermal gradient is induced to the sample plane at the center area of sample structure, and then, voltage is measured in a direction perpendicular to both the temperature gradient and the magnetization vector. 2) Variation of heating position by scanning pump laser toward or faraway the electrical contact did not alter the measured signal ΔV, as shown in Ref. 16 in the main text. 3) ΔV was obtained from the difference of magnetization-dependent signal between $+M$ and $-M$. We thought that normal Seebeck effect might affect the offset of the signal but not the magnetization-dependent signal in the longitudinal geometry with the local thermal excitation at the center, which cancels out most of lateral heat-diffusive effect. 4) Back-side illumination through transparent substrate of MgO clearly showed the inversion of the polarity of V/R curve as

shown in Fig. S2. 5) ANE itself was not enough to explain the measured data, as described in Fig. 3(b) in the main text.

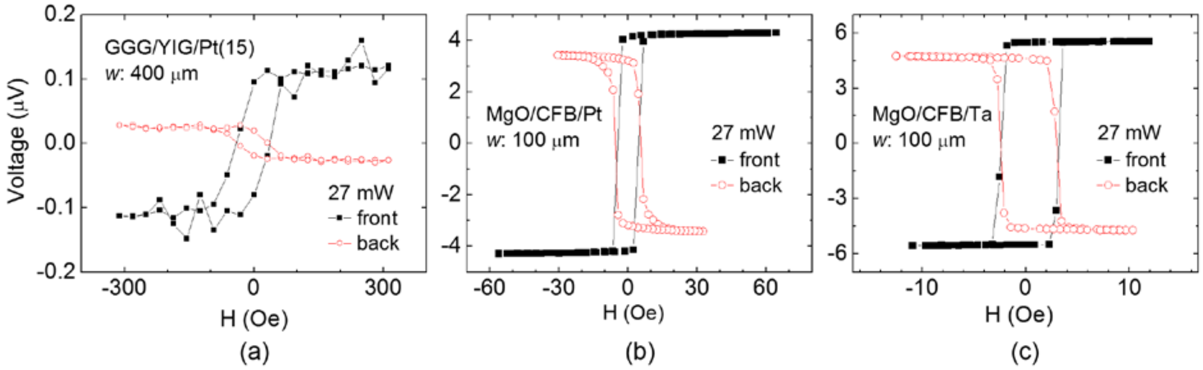

Figure S2 | Spin thermoelectric voltage dependence of GGG/YIG/Pt(15), MgO/CFB(8)/Pt(3), and MgO/CFB(8)/Ta(3) with front-side or back-substrate-side illumination with laser power of 27 mW.

Figure S2 illustrates the front-side or back-substrate-side illumation of laser beam to verify that the signal originates from vertical thermal gradient, which possibly induces ISHE in addition to ANE. The result of well-known YIG/Pt is shown in Fig. S2(a), when the sample is flipped around *y* axis, and then, measured the voltage along the conserved *y* axis. Resulting inverted curve of YIG/Pt implies that back-side pump imposes the reversed temperature gradient between FM and NM, thereby inducing ISHE with the opposite polarity. We could see the similar behavior in case of CFB/Pt or CFB/Ta. Note that the back surface quality of the film is different from the top surface.

∇T dependence on the optical absorption. Since we used the bulk value of thermal conductivity, the calculated value of C_{ANE} and S_S might be overestimated by considering the Eq. (2) in the main text, where for example, $S_S \propto V_{ISHE}/\nabla T_{FN}$. Interestingly, we found that an

absorption coefficient in the multilayer system is one of important parameters to affect the overall temperature gradient of the multilayer, as shown in Figs. S3 and S4. Absorption coefficients (α) were measured with 15-nm Ta, Pt, CFB samples, which revealed larger values than those of literature values, whereas the reflectivity showed smaller values, as shown in Table. S1. From this, we estimated that the effective absorption coefficient α^* lies within $\alpha/2 < \alpha^* < 2\alpha$ due to the combined effect of reflectivity and absorption. As described in Fig. 6 in the main text, this explains the rapid increase of Δ*V*, when the number of repeat (*n*) is less than 3~4.

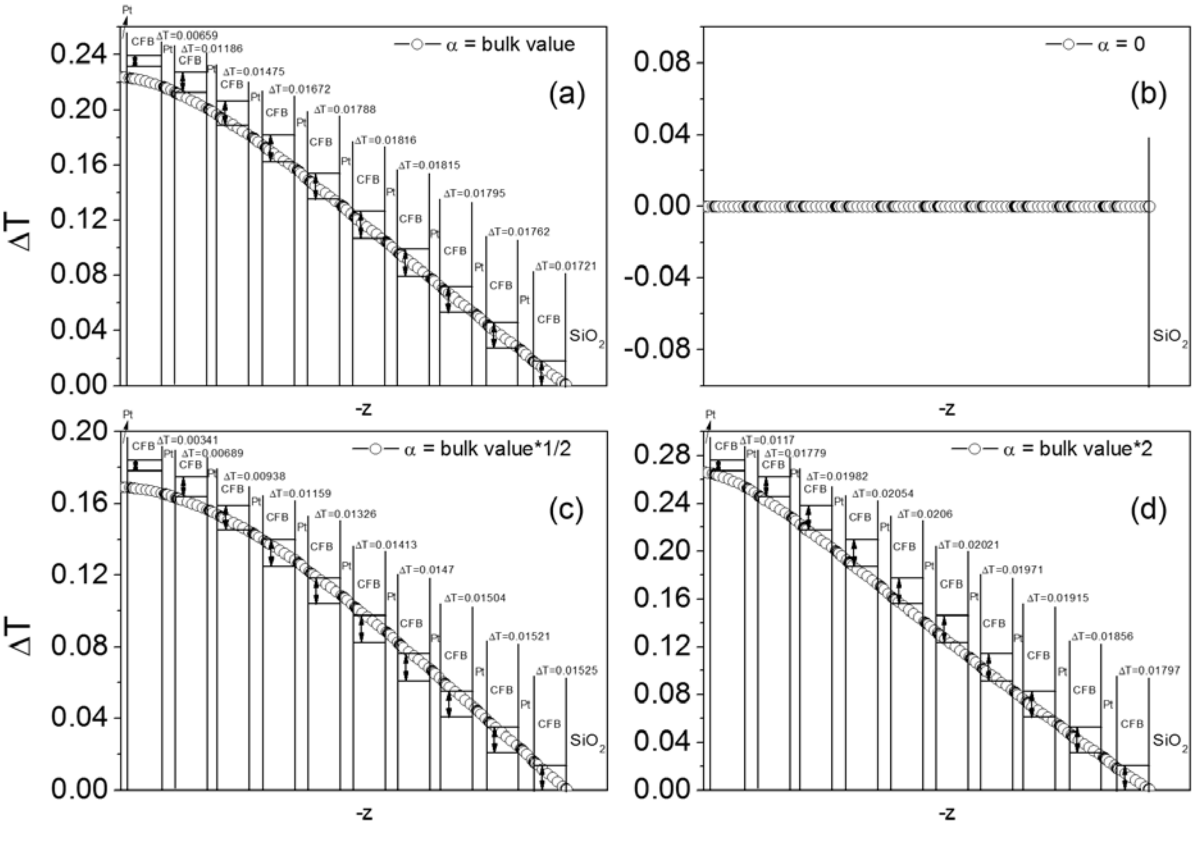

Figure S3 | Δ*T* (≡ *T* - *T*(bottom)) is shown as a function of *z*. Absorption coefficient α is varied from 0 to bulk value × 2 to see its dependence on Δ*T*.

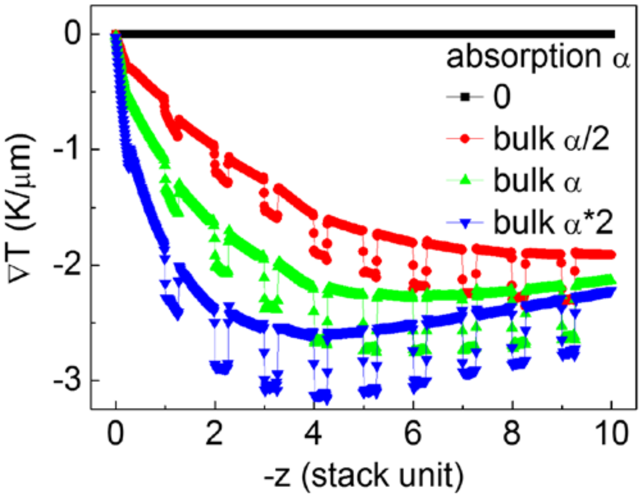

Figure S4 | ∇T (≡ dT/dz) is shown as a function of z in stack unit.

Table S1 | Reflectivity and absorption coefficent of thin film (15 nm) of Ta, Pt, CFB.

	reflectivity (bulk)	α (bulk, 10^5/cm)	reflectivity (15 nm)	α (15 nm, 10^5/cm)
Ta	0.74	5.54	0.45	8.14
Pt	0.71	7.78	0.59	8.59
CFB	0.7	4	0.40	7.90

We would like to emphasize that ΔT_F of CFB in Fig. 3(e) in the main text did not alter much by varying the thermal conductivity of Pt or Ta due to the top-to-bottom optical injection geometry, in which effect of the lateral thermal conduction through the thin film on the signal is mostly cancelled out. It depends mostly on the absorption coefficient and thermal conductivity of top-layer CFB. Therefore, we think that strong evidence for the existence of inverse spin Hall effect is Figs. 3(b) and (e), which reveals nearly identical ΔT_F of CFB in Fig. 3(e), so almost same ANE, but quite different level of V/R curve in Fig. 3(b). Qualitatively, the reason of negative sign of Ss of Ta stems from low $\Delta V/R$ but high ΔT_{Ta} compared with high $\Delta V/R$ but low ΔT_{Pt} as depicted in Figs. 3(b) and (e). Relatively high ΔT_{Ta} is related to the high electrical resistivity, low thermal conductivity, and not much different optical absorption

coefficient of β-Ta compared with Pt. If we consider the lower thermal conductivity by using Wiedemann-Franz law of metals like CFB, Pt, and (β-)Ta, this tendency becomes clearer due to more distinctive thermal gradient. From these reasons, we think that the variation of thermal conductivity of bulk value to thin film value (estimated by Wiedemann-Franz law; S. Yoneoka *et al.*, Nano Lett. **12**, 683 (2012)) does not alter our main point, which reveals the negative sign and non-ignorable value of Ss of Ta.

Estimation of figure of merit. With the analogy of Eq (2) in the main text to the thermomagnetic figure of merit as described in the equation (1.28) of Ref. 1, the effective thermomagnetic power S_m can by estimated by

$$S_m \sim dV/dT \sim \Delta V/(\nabla T \times d), \quad (3)$$

where ∇T is vertically-induced average temperature gradient, and d is the diameter of the beam spot. For a single stack of CFB(15)/Pt(15) when w = 0.4 mm and d = 80 μm, $\Delta V \sim$ 0.382 μV. Since $|\nabla T_F| \sim |\nabla T_{FN}|$ in this case, we used the average temperature gradient by calculating $\nabla T \sim (|\nabla T_F| + |\nabla T_{FN}|)/2 \sim 7290$ K/m. Combined bulk thermal conductivity was calcuated by $\kappa_\perp \sim 2\kappa_F\,\kappa_N/(\kappa_F + \kappa_N)$, where $\kappa_\perp$ is the thermal conductivity in the vertical direction. Likewise, the effective electrical conductivity along the sample plane was calculated by $\sigma_{||} \sim (1/\rho_F + 1/\rho_N)/2$, where ρ_F and ρ_N are the measured resistivity of the FM and NM layer, respectively. From these, when T = 300 K, we roughly estimated $S_m \sim 6.55\times10^{-7}$, and $ZT \sim \sigma_{||}\, S_m^2\, T/\, \kappa_\perp \sim 0.57\times10^{-5}$.

By comparing the ratio of the power factor $\sigma_{||}\, S_m^2$, we can further estimate $(ZT)_{n,w}$ of multilayer with different sample width. The increase of the number of stack repeat from 1 to 10 and the reduction of the sample width from 100 to 10 μm are compensated each other in terms of $R_{||}$, so, the effect of $\sigma_{||}$ is unchanged. When we assume the same effective thermal conductivity of $[CFB/Pt]_i$, where i = 1 ~ 10, the ratio of $(ZT)_{n,w}$ solely depends on the ratio of

S_m^2. Since ΔV (∇T) of CFB(8)/Pt(3) with width of 100 μm is 0.004 mV (0.74 K/μm; see Fig. 6 in the main text), and ΔV (∇T) of $[CFB(8)/Pt(3)]_{10}$ with width of 10 μm is 0.21 mV (2.29 K/μm; maximum value is used for under-estimation). Hence, we roughly calculated the ratio of the power factor with ~302.0, and then, $(ZT)_{n=10,w=10\mu m}$ ~ ZT×302.0 ~ 1.73×10^{-3}.
